# Homodyne detection of short-range Doppler radar using a forced oscillator model

**DOI:** 10.1038/srep43680

**Published:** 2017-03-02

**Authors:** Kunanon Kittipute, Peerayudh Saratayon, Suthasin Srisook, Paramote Wardkein

**Affiliations:** 1 Department of Telecommunication Engineering, Faculty of Engineering, King Mongkut’s Institute of Technology Ladkrabang, Ladkrabang, Bangkok 10520 Thailand; 2 Defence Technology Institute, Ministry of Defence, BanmaiPakkret, Nontburi 11120 Thailand

## Abstract

This article presents the homodyne detection in a self-oscillation system, which represented by a short-range radar (SRR) circuit, that is analysed using a multi-time forced oscillator (MTFO) model. The MTFO model is based on a forced oscillation perspective with the signal and system theory, a second-order differential equation, and the multiple time variable technique. This model can also apply to analyse the homodyne phenomenon in a difference kind of the oscillation system under same method such as the self-oscillation system, and the natural oscillation system with external forced. In a free oscillation system, which forced by the external source is represented by a pendulum with an oscillating support experiment, and a modified Colpitts oscillator circuit in the UHF band with input as a Doppler signal is a representative of self-oscillation system. The MTFO model is verified with the experimental result, which well in line with the theoretical analysis.

In the self-oscillation system, the amplitude of the oscillator in steady state was independent from initial state^1^, and this steady amplitude can be determined in the term of the compensation between the transferred energy which produced by nonlinear part of system, and energy losses which occur in all practical systems. Thus, if the external source was applied through the system, the external source would be defined as a perturbed function in nonlinear part of the system, or we can interpret this nonlinear part as the modulation part between a feedback of the oscillation with the external forced. Resulting, general system modelling is based on the operation of each circuit element, has been emphasised, such as feedback analysis and specific modelling. These are impressive and causal methods for explaining the system response, such as different circuit structures producing different system responses^2^,^3^,^4^,^5^,^6^,^7^,^8^,^9^,^10^,^11^,^12^,^13^,^14^,^15^,^16^,^17^,^18^,^19^,^20^,^21^,^22^,^23^,^24^,^25^,^26^,^27^,^28^,^29^,^30^,^31^,^32^,^33^,^34^,^35^,^36^,^37^,^38^,^39^,^40^,^41^,^42^,^43^,^44^,^45^,^46^,^47^. For example, self-excited electrical oscillator cases have been based on Adler’s equation^2^,^3^,^4^,^5^,^6^,^7^ or the Van der Pol equation^8^,^9^,^10^,^11^, therein being combined with perturbation methods, which depend upon many complementary factors, including the physical characteristics of the system and the system’s elements and boundary conditions^12^,^13^,^14^,^15^,^16^. Moreover, phase-domain analysis has been used in some studies^17^,^18^,^19^. In the short-range radar (SRR) circuit case, the Symbolical Abbreviated Equation (SAE) which based on perturbation method was playing an important role to analyse an oscillator which simultaneously generates the transmitted signal and modulates the transmitted and reflected signal, this self-oscillator is called autodyne^9^. This model was appropriatefor in-depth analysis of specific feature in system, such as the effect of the external impact with definite time delay^20^, the characteristics of noise in autodynes^21^.

Current military technology has applied SRR to many disciplines, such as muzzle velocity measurement, short-range air electronic counter measure, and proximity fuzes^22^,^23^,^24^,^25^. The Doppler SRR is a continuous-wave radar which can be categorized into two types, the first is unmodulated continuous-wave radar and second modulated continuous-wave radar. The unmodulated continuous-wave radar can operate by sending out a transmitted signal, which is later reflected by a target. Here, the reflected signal will possess a shift in frequency that directly varies with the speed of the target itself. Such a phenomenon is called the Doppler effect, hence the name “Doppler frequency”^26^.

Among the many techniques for recovering the Doppler frequency in the unmodulated continuous-wave radar, the directly one is homodyne detection, which can directly convert the Doppler-shifted frequency into the Doppler frequency. Homodyne detection is a method of detecting the desired signal by multiplying the reference signal (the local oscillator) and the arbitrary signal (the shifted signal, or the modulated signal). Then, the homodyne technique can be used as one of the important parts of many types of science and engineering experiments^27^,^28^,^29^,^30^,^31^,^32^,^33^,^34^,^35^,^36^,^37^,^38^,^39^,^40^,^41^,^42^,^43^,^44^,^45^,^46^,^47^,^48^. For instance, to verify quantum entanglement, the homodyne technique was used to detect a variant of the quantum states^27^,^28^,^29^,^30^,^31^,^32^,^33^,^34^,^35^,^36^,^37^,^38^, which entangle each other. In gravity wave detection, homodyne detection was an important key in laser interferometry for detecting the gravitational signal^39^,^40^,^41^,^42^,^43^,^44^,^45^,^46^. Conventionally, the homodyne in self-oscillation system was defined as self-mixing oscillator, which can be analysed with perturbation method, where the amplitude of the self-oscillation system only depends on a nonlinear damping in the steady state.

However, the evidence for the dependence between the initial condition and self-oscillation system was reported in T. Maneechukate *et al*.’s research^47^,^48^. They develop the multi-time forced oscillator (MTFO) model with the signal and system framework, this model consists of signal and system theory, a second-order differential equation, and the multiple time variable technique. As a result, a greater number of frequency components can be explained. In the signal and system background, arbitrary physical quantities are the information, as depicted by the signal, and the external force is shown by the input signal, with these signals transformed into an output signal from the system. These output signals are the consequence of interaction between an external force and the system; we call this the signal and system theory^49^,^50^. In this principle, almost any physical system can be modelled by a differential equation that describes the relationship between the output and input quantity and that represents the law of motion of the system, such as a mechanical system based on Newton’s law, an electrical circuit based on Kirchhoff’s law, or the laws of the circuit’s constitutive elements. From this point of view, arbitrary structure of forced oscillation system based on same mathematical tool, the second order differential equation, and noted that the nonlinear behaviour is a basis pattern for all kind of forced oscillating structure. Then MTFO model was the simple model to analysed a self-oscillation system with a forced system perspective, which constructed from a second order differential equation. The complete solution composed of two independent solution (homogeneous and the particular solutions) and the independent time variable for each solution, with multi-time variable initial condition, which interpreted as the amplitude of natural response effected by forced response at any time, not just at the initial time. Thus, the origin of nonlinearity of system can be derive from this interpretation (more detail in result section). The advantage of MTFO model is applying to every forced oscillating system analysis, without constrained of specific structure (such as mechanical and electrical system), then we can apply MTFO model to analyse any kind of scheme in homodyne detection device. In this article, homodyne in a forced oscillator background was observed to detect the Doppler signal. We analyse a single forced oscillator circuit applied in an SRR system with the MTFO model. The MTFO model represents another way of looking at such phenomenon from the simple viewpoint of signal and system theory: an oscillator circuit under its forced oscillation state, where the Doppler-shifted signal is the forced input. This is the difference between MTFO model and conventional model, the general electrical oscillator was classified as the self-oscillation system.

Here, a Doppler SRR circuit is considered to be similar to an oscillator that is forced by an external input, and the homodyne detection occurring from the phenomenon has been explained using the MTFO model. Apart from this, homodyne detection in the Doppler SRR circuit has been discovered. A low-frequency component was then found and singled out, which result was similar to self-mixing oscillator^51^. Furthermore, the analysis under signal and system theory enables the feeding of a forced input, or simply a Doppler-shifted signal, into the circuit that can also be performed at any point, which implies that the system response to the external source are all the same under the same principle.

## Result

### MTFO model

For in-depth understanding, a forced-input oscillation shall be explained using this signal and system first with a conventional analysis and then with the MTFO model.

In general, the explanation of an oscillator circuit under a single tone sinusoidal forced input situation can be accomplished using a second order differential equation to find a natural response *x*_*n*_(*t*) in Eq. (1). While a forced response is named a sinusoidal steady state response *x*_*f*_(*t*) shown in Eq. (2). A complete response *x*_*o*_(*t*) is composed of both natural and forced responses according to Eq. (3).


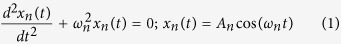



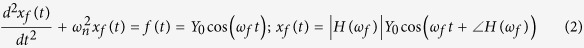



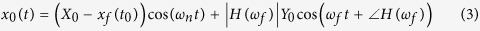


where *A*_*n*_ is the natural response’s amplitude, which depends upon the initial condition of the system *X*_0_ and the forced response at initial time *x*_*f*_(*t*_0_); *ω*_*n*_ is the natural frequency generated from the oscillator; *Y*_0_ is the magnitude of the input fed into the system; *ω*_*f*_ is the input frequency; |*H*(*ω*_*f*_)| is the magnitude response of the transfer function of an oscillator *H*(*ω*_*f*_); and ∠*H*(*ω*_*f*_) is the phase response of the oscillator’s transfer function. From the complete response in Eq. (3), the amplitude of the natural response is affected by the forced response at the initial time only, which is in contrast with experimental results.

Recently, T. Maneechukate *et al*. used a second-order differential equation with the multiple time variable technique to solve a variety of forced oscillator problems using a single model^47^,^48^,^52^,^53^,^54^,^55^,^56^,^57^, the MTFO model. The results of their experiment confirmed that the forced response had an effect on the amplitude of the natural response at any time. To simplify analysis, we set *t*_0_ = 0, the amplitude of the natural response in MTFO model can be shown as Eq. (4).


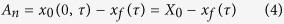


Then the complete response based on MTFO model can be shown as Eq. (5)


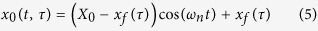


where *τ* is an arbitrary time variable, we can also interpret *τ* ≡ *t* − *t*_0_ as a time delay, with *t*_0_ is the initial time of the forced oscillation system. We can note that the term of 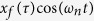 from (5), is the occurrence for nonlinearity of the system.

### Homodyne detection in a forced oscillation system

From our perspective, the Doppler shift signal reflected from a moving target as the input into the oscillator is shown in Eq. (6), and the resulting forced response of the second-order oscillator system can be found as shown in Eq. (7). Substituting this value into Eq. (5), one will obtain an absolute response of the second-order oscillator as shown in Eq. (8).


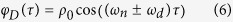



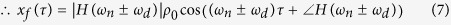



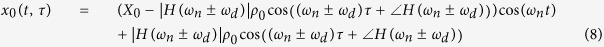


where *ρ*_0_ is magnitude of the reflected Doppler, and *ω*_*d*_ is the Doppler frequency.

To make it easy to understand, it is therefore assumed that *t*_0_ = 0, and then *τ* ≡ *t*. When expanding Eq. (8), one can rewrite a new complete response as shown in Eq. (9).


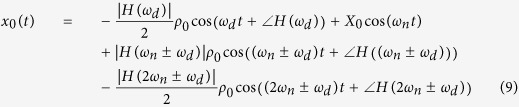


It can be observed that the first term of Eq. (9) is the explicit Doppler frequency. By passing this absolute response through a low-pass filter, one can recover the Doppler signal without using an AM demodulator.

**Figure 1:**
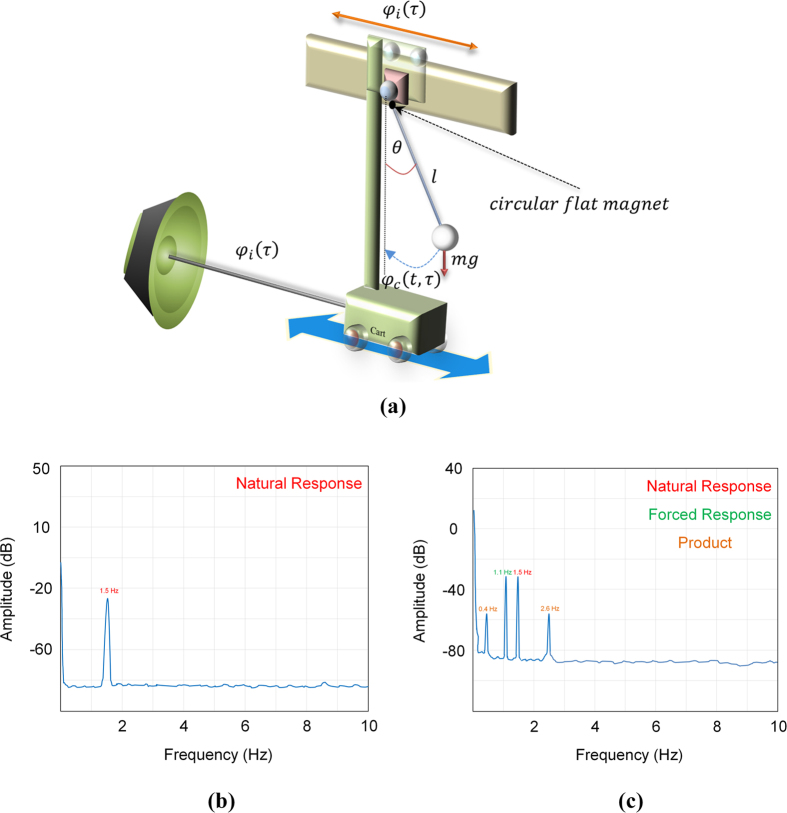
Pendulum with an oscillating support experiment. (**a**) A light rod 0.1 m in length is attached to a mass of 0.1 kg at one end and to a rotating point at the other end at the movable support; near this one end of the light rod, a circular flat magnet is attached. This movable support is directly connected to the cart’s pillar, and the body of the cart is connected to the centre of the speaker, which is controlled by a signal generator. Thus, the output signal from the speaker is the external input *φ*_*i*_(*t*) that is injected through the support of the pendulum system. The light rod’s position *φ*_*c*_(*t, τ*) is measured by a Hall effect motion sensor, UGN3503, that is placed near the circular flat magnet; the sensor is connected through an oscilloscope, and the FFT (fast Fourier transform) function is used to display the output in the frequency domain. Moreover, the pendulum swings less than 5 degrees to avoid nonlinear behaviour. (**b**) A natural frequency spectrum at 1.5 Hz, with no forced input from the oscillating support. (**c**) Complete response spectra of the forced pendulum system, which is forced by a sinusoidal wave at 1.1 Hz.

From the above, a simple mathematical analysis with an obvious homodyne detection in the arbitrary forced oscillation system has been shown, and the MTFO model was confirmed by a different structure of the forced oscillation system in T. Maneechukate *et al*.’s articles. The interesting experiment from their article that has inspired us was the pendulum with an oscillating support experiment^54^, where homodyne behaviour in the mechanical oscillation system occurred which based on the MTFO model. We redrew the experiment and results from their article in Fig. 1. While the forced was switched-off, the pendulum system was a free oscillation system, which their natural response *φ*_*n*_ (*t*) can be explained by Eq. (11). By define *g* is the gravity acceleration constant, and *l* is the length of light rod. The natural frequency of pendulum system can be obtained as 
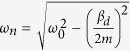
, by 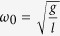, their amplitude would decay respect to time, due to damping constant *β*_*d*_.


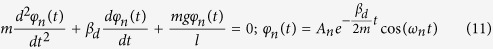


We can note that this system is not a self-oscillation system, due to it can oscillate without the external source. After we apply the external forced 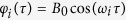, the forced response *ϕ*_*f*_(*τ*) was found as Eq. (12).


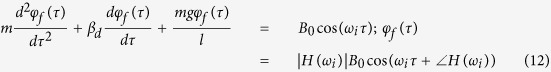


where magnitude and phase response can be obtained in Eq. (13).


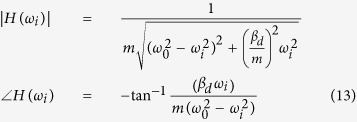


The amplitude of natural response *A*_*n*_ can be derived as Eq. (4), then the complete response *φ*_*c*_(*t, τ*) of this forced oscillation system can be shown as:


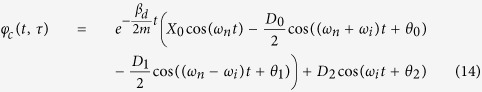


where *D*_0_ = *B*_0_|*H*(*ω*_*n*_ + *ω*_*i*_)|, *D*_1_ = *B*_0_|*H*(*ω*_*n*_ − *ω*_*i*_)|, *D*_2_ = *B*_0_|*H*(*ω*_*i*_)|, *θ*_0_ = ∠*H*(*ω*_*n*_ + *ω*_*i*_) − *ω*_*i*_*t*_0_, *θ*_1_ = ∠*H*(*ω*_*n*_ − *ω*_*i*_) − *ω*_*i*_*t*_0_, and *θ*_2_ = ∠*H*(*ω*_*i*_) − *ω*_*i*_*t*_0_.

From the results, the direct conversion achieved by forcing the pendulum with a sinusoidal signal is based on the MTFO model. These results implied that there is intrinsic homodyne for an arbitrary oscillation system; therefore, the MTFO model can also be used for specific structures, such as a modified Colpitts oscillator.

### Modified Colpitts oscillator analysis using the MTFO model

**Figure 2:**
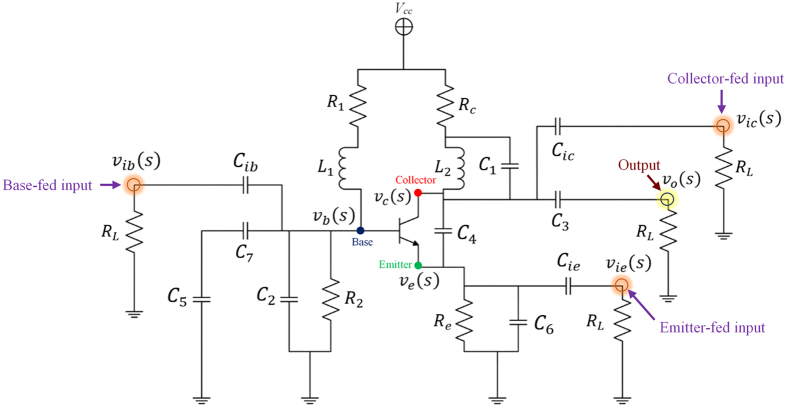
Radar circuit. Modified Colpitts oscillator circuit in our experiment.

In this section, more potential for using MTFO has been shown. The complicated of many difficult equation is raised due to more complex system analysed by basic dynamic law with multi-time variable technique only, otherwise the second order within higher order differential can be shown in this section, then the MTFO model still can be applied to analysed system. Then a second-order oscillator can be used for homodyne detection, as demonstrated in this article using a modified Colpitts oscillator in Fig. 2.

**Figure 3:**
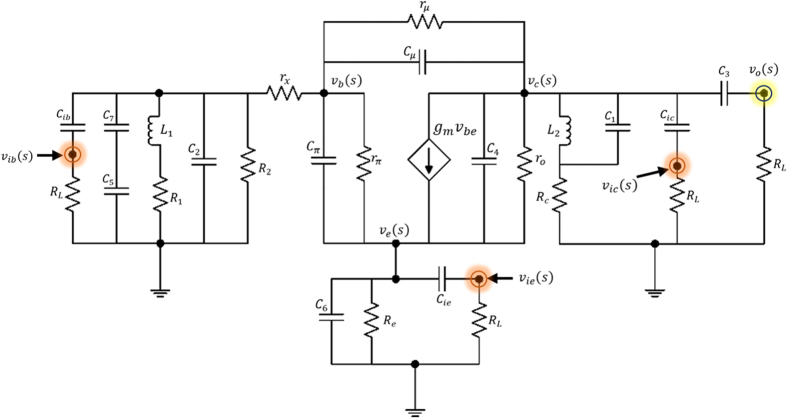
Equivalent circuit. High-frequency small-signal equivalent circuit of a modified Colpitts oscillator.

**Figure 4:**
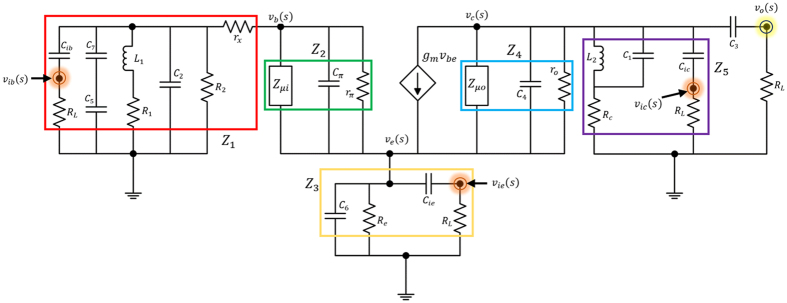
Equivalent circuit with reduction. High-frequency small-signal equivalent circuit of modified Colpitts oscillator after circuit reduction.

To prove homodyne detection in the modified Colpitts oscillator, which is based on the MTFO model, one can analyse the system by starting from law of dynamic of the circuit such as KCL (Kirchhoff current law), when there is still no Doppler reflected from the target, and find the natural frequency response. Here, a high-frequency small-signal equivalent circuit^58^ of the circuit in Fig. 2 is shown in Fig. 3, where *C*_*π*_, *r*_*π*_*C*_*μ*_, and *r*_*μ*_ are intrinsic capacitor and resistor which depend on active BJT device. When using Miller’s theorem^58^ to separate *C*_*π*_ and *r*_*π*_ to be *Z*_*μi*_ and *Z*_*μo*_, one can rewrite a new equivalent circuit as shown in Fig. 4. After applying KCL (Kirchhoff current law)^59^,^60^ into each node and using Laplace transform, the following relationships can be found as:


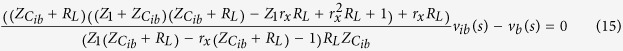



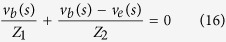



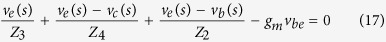



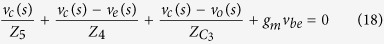



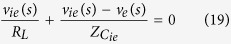



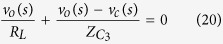



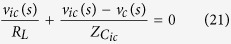


where *v*_*ic*_(*s*), *v*_*ib*_(*s*), and *v*_*ie*_(*s*) are the collector-, the base-, and the emitter-fed input node respectively. The BJT ports node for the collector, the base, and the emitter are represented as *v*_*c*_(*s*), *v*_*b*_(*s*), and *v*_*e*_(*s*). And *v*_*o*_(*s*) is the output node. And the iedance for *Z*_1_, *Z*_2_, *Z*_3_, *Z*_4_, and *Z*_5_ can be found as:


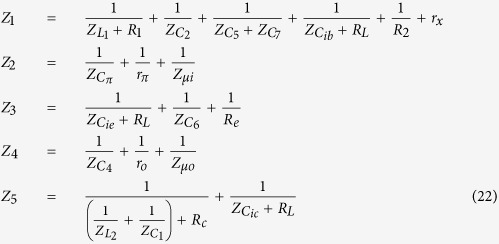


By 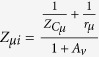 and 
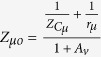
 with *A*_*v*_ is the collector to emitter gain. When substituting the impedance of any capacitors with 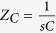 and the impedance of any inductance with *Z*_*L*_ = *sL*, one can solve Eqs (15) to (21) with zero input having been set (*v*_*ib*_(*s*), *v*_*ie*_(*s*), *v*_*ic*_(*s*) = 0). The homogenous equation of the modified Colpitts oscillator can be shown in Eq. (23).


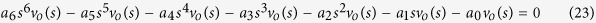


where *a*_6_, *a*_5_, *a*_4_, *a*_3_, *a*_2_, *a*_1_, and *a*_0_ are simplify parameters (see Method).

After solving Eq. (23), the natural frequency is derived as in Eq. (24).


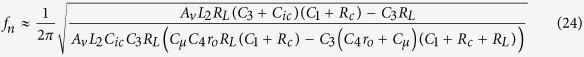


A suitable point where one should feed the reflected Doppler is not necessarily through a system feedback path, the output of the circuit. For an oscillator under forced oscillation, an analysis can be performed when an input signal is injected into either the emitter, the collector, or the base of the transistor, while the output can be measured at the Collector. In this article, an experiment and analysis had been undertaken for all cases where the reflected Doppler has been injected into the emitter, the collector and the base of the transistor.

As the reflected Doppler has been injected into the base circuit of the transistor of the oscillator, the analysis must be carried out on the transfer function of the system. By solving node equations, the relationships between the base, emitter, or collector inputs and the output are shown in Eqs (25) to (27), respectively.


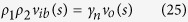



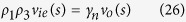



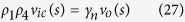


where


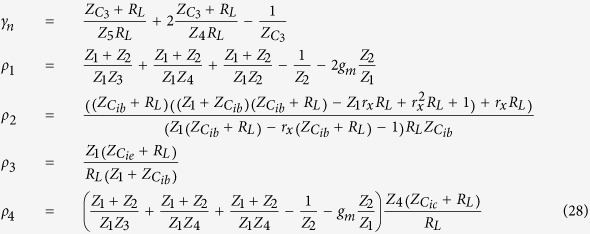


After substituting the impedance value into the Eqs (25) to (27) and taking some approximation by omitting some insignificant terms, the magnitude and phase response of the system can be found as shown in Eqs (29) to (31).


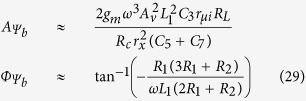



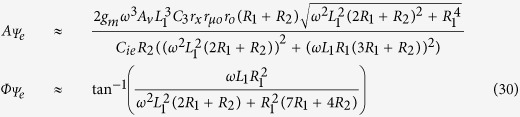



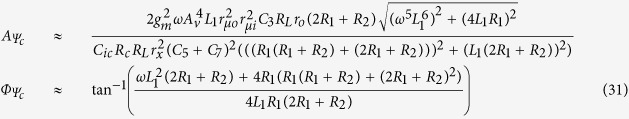


Subsequently, the forced response of the system for the base-, emitter-, and collector-fed circuits can be found by substituting Eqs (29) to (31) into Eq. (6) as follows:


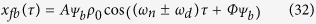



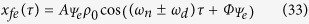



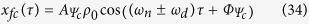


From the above analysis, a complete response for the base-, emitter-, and collector-fed circuits has been established as shown below:


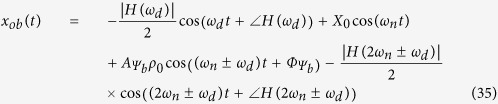



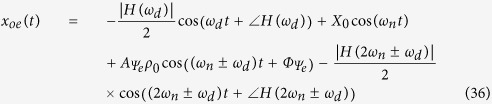



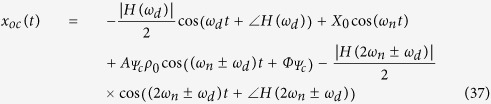


From the above analysis of the forced response, it can be concluded that the reflected signal can be fed into either the collector-, the base-, or the emitter-fed circuits of a transistor. Additionally, when substituting into the absolute response, the result will be similar. Only the magnitudes and phases will differ; however, in all cases, the first term obtained will be the Doppler frequency component. From these, we can apply more than one Doppler shifted signal to circuit, and we still also can classify the point of fed circuit by magnitudes and phases response of system. Then, we can apply this advantage to improve more application in our future work, such as define multiple target in three different direction. In next section, these system analyses have been investigated by our experiment.

### Short-range Doppler radar experiment

**Figure 5:**
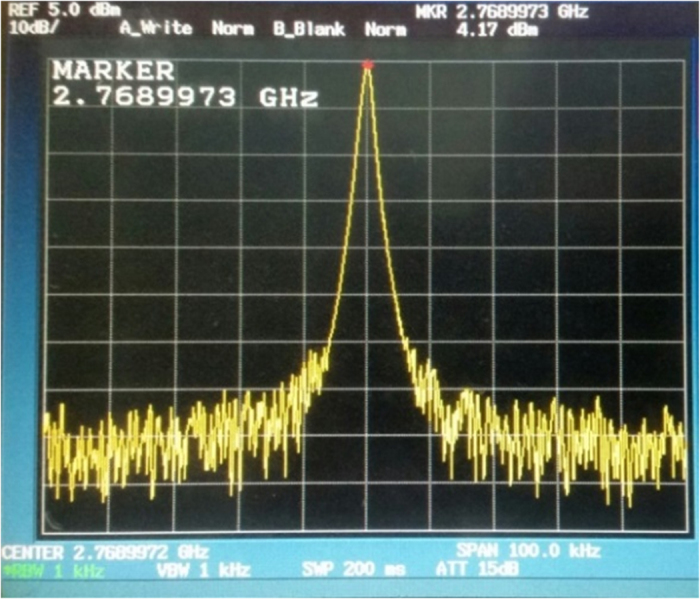
Natural frequency. Before applying a Doppler-shifted signal to the oscillator, the spectrum of the natural frequency was measured at 2.76 GHz.

From the previous section, we verify the principle by an experiment using a modified Colpitts oscillator that was established from the schematic in Fig. 2. While there is not any Doppler reflected back, the spectrum of the output signal from the oscillator measured with a spectrum analyser set at a normalized impedance value of 50 Ω is shown in Fig. 5. The signal obtained is a sinusoidal signal with a fundamental frequency of 2.76 GHz.

Next, a reflected Doppler was fed into the oscillator circuit, and all possibilities, namely the emitter, the collector, and the base, were used to receive the input. From our experimental observation, the sensitivity of this modified Collpitts oscillator, without add on processing process is −57.13 dB with range of bandwidth 40 kHz. Thus, an imitating Doppler-shifted signal from an RF generator equivalent to a signal reflected from a target with a relative velocity of 3 Mach at a distance of 5 m is used (equivalent to the distance in an idealistic experiment of 10 m), which is approximately equal to 20 kHz. From such a distance, the free-space path loss attenuation is calculated as −61.3 dB.

**Figure 6:**
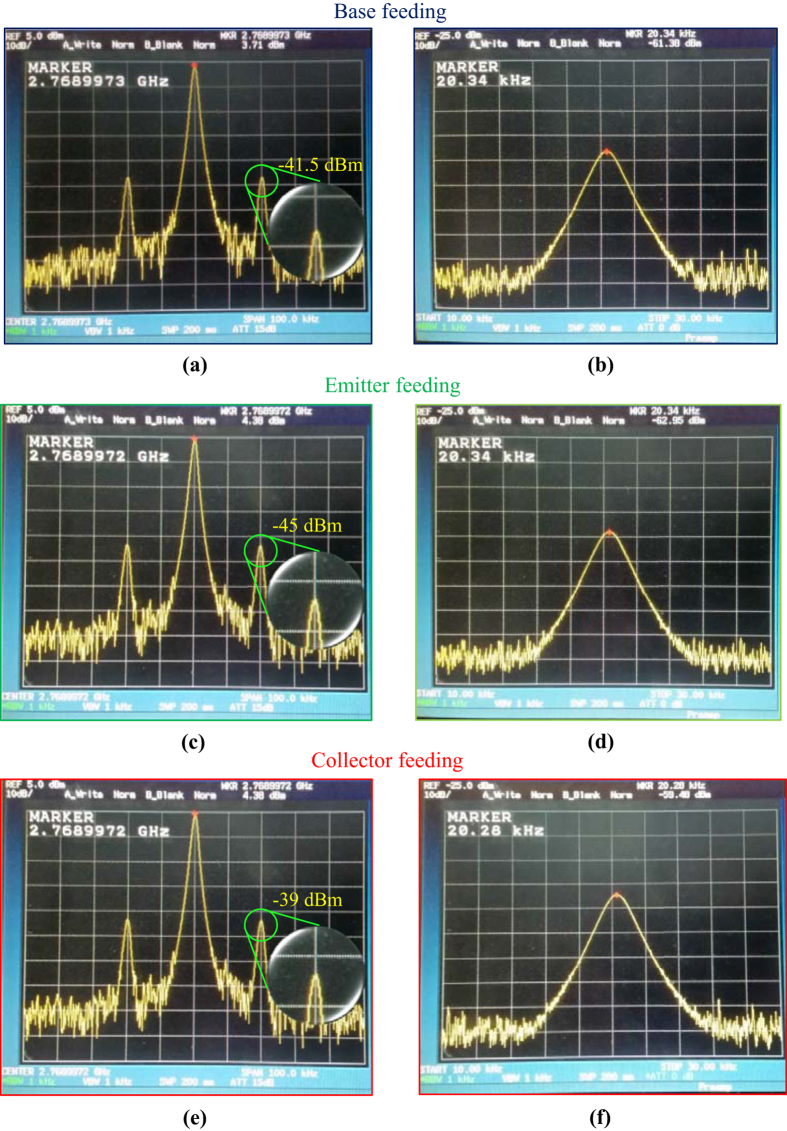
Output spectrum of the modified Colpitts oscillator. Output spectrum in a natural frequency band and a Doppler frequency band when feeding Doppler-shifted signal into the base (the spectra are shown in **(a,b**)), the emitter (the spectra are shown in **(c**,**d**)), and the collector (the spectra are shown in (**e**,**f**)) of the transistor.

When feeding the Doppler-shifted signal into the base-, emitter-, and collector-fed circuits, the resulting output from the collector of the transistor appears in the radar frequency spectrum as an AM signal which similar to autodyne signal as shown in Fig. 6(a,c,e), respectively. The output spectrums of the Doppler are as shown in Fig. 6(b,d,f), respectively. The results presented in Fig. 6 show that the oscillator sensitivity essentially depends upon the feed-point of the Doppler-shifted signal. The more noteworthy from Fig. 6 is the low amplitude of Doppler signal, which compare with the amplitude of natural response and the Doppler shifted signal amplitude. Then the Doppler signal hard for investigate directly in time domain, but it still exists in frequency domain observation.

## Discussion

This article has been presented a new method of homodyne detection by using the MTFO model which based on the forced oscillation perspective, where can applied in a Doppler SRR. The analysis is based on signal and system theory, a second-order differential equation, and the multi-time variable technique. The mathematical technique of MTFO is uncomplicated and can also be applied to self-oscillation system such as electrical oscillator or arbitrary structure of oscillator. This imply that the forced oscillating behavior is the intrinsic behavior for every kind of the oscillator, even self-oscillation system. Then we also can apply to another different homodyne detection in the future, such as optical device. Otherwise, the nonlinear model still be an appropriate choice for analyse in-depth characteristic in self-oscillation system. From results, the output of natural oscillation system with external forced still has some difference from self-oscillation result. The AM signal or autodyne signal which occur from the output in radar frequency band is not appear in a forced pendulum’s result, and this autodyne signal can be obtained from SAE method only. Although, the MTFO model is not suitable for profound analysis, but it can explain the different kind of oscillation system under single principle, then the MTFO model is the other interesting choice to analyse primary phenomenon, homodyne detection, in all of oscillation system. This phenomenon has been observed in the free oscillation system which forced by external source such as pendulum with an oscillating support experiment, and same behaviour in the self-oscillation system, which has been confirmed experimentally using a RF generator feeding a simulating reflected signal into a modified Colpitts oscillator, which generates a 2.76 GHz output. The experimental results show that the Doppler-shifted signal can be fed into any inputs of a transistor and not strictly into the feedback path of circuit. However, the Doppler output will be most prominent, and hence the system response will be highest when feeding a reflected signal into the collector of the transistor, followed by base feeding and then emitter feeding. Furthermore, the result is in line with the analysis, which confirms that there is a Doppler frequency component in the lower side of the spectrum and that the forced input can be applied into any terminal of a transistor. Moreover, the difference of magnitude and phase response for each fed circuits, the directional finding in three direction can be applied in the future.

## Methods

### Circuit Component

The modified Colpitt oscillator was made using the schematic in Fig. 1, where the values of the components used are as follows: *R*_1_ = 5.6 *k*Ω; *R*_2_ = 1 *k*Ω; *R*_*e*_ = *R*_*c*_ = 10 Ω; *L*_1_ = *L*_2_ = 15 *μH*; *C*_1_ = 15 *pF*; *C*_2_ = 55 *pF*; *C*_3_ = *C*_4_ = *C*_5_ = 3.3 *pF*; *C*_6_ = 27 *pF*; *C*_7_ = 1 *pF*; *C*_*ie*_ = *C*_*ib*_ = *C*_*ic*_ 3.3 *pF*; *V*_*cc*_ = 12 *V*. And a transistor in this circuit is BFG135.

### Experimental Setup

**Figure 7:**
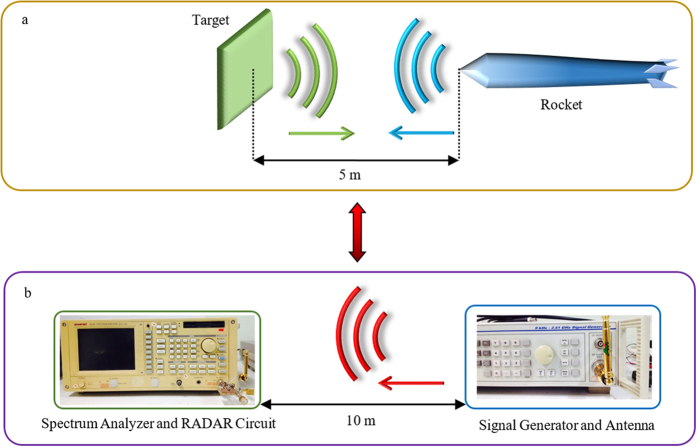
Equivalent experimental setup. (**a**) Real situation set as a rocket is moving forward to the rectangle target with a 5 m distance, equivalent to the idealistic experiment in (**b**). (**b**) The idealistic experiment, where the transmission and reflection distance were gathered.

For the explicit application proposed, a real situation was examined when a proximity fuze was 5 m away from a flat rectangle target (area ≈ 0.328 m^2^) with a relativistic velocity of 3 Mach. All loss budgets in this short-range RADAR situation were calculated using the RADAR equation (Eq. (38)). Due to the transmission frequency being close to the reflection frequency, we then approximate them to be equal. We establish the idealistic equivalent by using an RF generator with a monopole antenna of 5.19 dBi located 10 m away from the circuit to gather the transmission and reflection distance, as shown in Fig. 7. The power of the generated RF signal (or Doppler-shifted signal) equals the output power compensated by the antenna gain for −5.19 dBc.


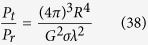


where *P*_*t*_ is the transmit power, *P*_*r*_ is the receive power, *R* is the distance between the proximity fuze and the target, *λ* is the wavelength of the carrier signal, *G* is the antenna gain, and *σ* is the RADAR cross section of the target. From (34), given *R* is 10 m, we can calculate *σ* ≈ 115.14 m^2^, and the Free Space Path Loss attenuation is calculated as −61.29 dB.

**Figure 8:**
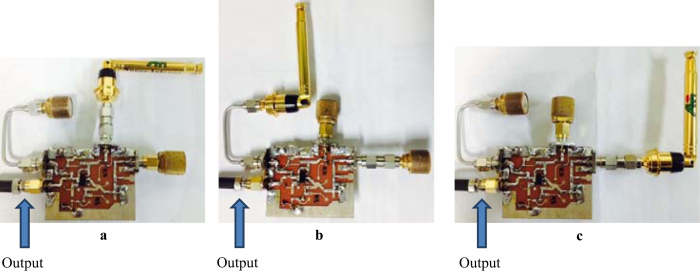
UHF modified Colpitts oscillator circuit for the experiment. (**a**) Emitter-fed circuit. **(b)** Collector-fed circuit. **(c)** Base-fed circuit.

In the oscillator circuit, an equivalent input with a simulated Doppler shift is fed through the antenna connected to the impedance-matching circuit, which is attached to the selected transistor port. For the other terminals, dummy loads were connected with impedance-matching circuits as shown in Fig. 8.

### Simplify parameters

To avoid unnecessarily complicated for circuit analysis, we define parameters as list below:


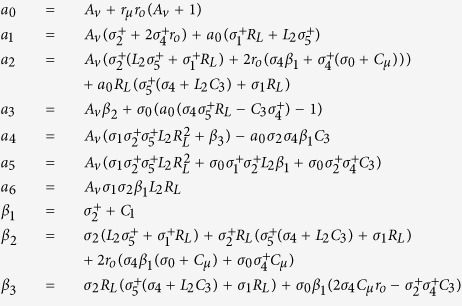



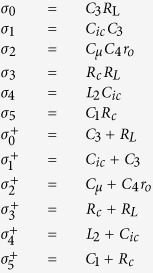


## Additional Information

**How to cite this article**: Kittipute, K. *et al*. Homodyne detection of short-range Doppler radar using a forced oscillator model. *Sci. Rep.* **7**, 43680; doi: 10.1038/srep43680 (2017).

**Publisher's note:** Springer Nature remains neutral with regard to jurisdictional claims in published maps and institutional affiliations.
